# How to systematically and quantifiably remove meaning?

**DOI:** 10.3389/frai.2026.1783410

**Published:** 2026-05-13

**Authors:** Frida Proschinger Åström, Arend Hintze

**Affiliations:** 1 Data Analytics, School of Information and Engineering, Dalarna University, Falun, Sweden; 2 BEACON Center for the Study of Evolution in Action, Michigan State University, East Lansing, MI, United States

**Keywords:** large language models, meaning and semantics, meaning degradation, robustness evaluation, semantic erosion

## Abstract

Large language models increasingly mediate real-world tasks, yet we lack systematic ways to quantify how their performance degrades when the meaning of their inputs is eroded. To bridge this gap, we developed a framework to semantically erode meaning and quantify its intensity, grounded in discourse analysis, psycholinguistics, and software engineering, comprising five theoretically motivated methods: omission of key information and context, lexical substitution with near-synonyms, increased abstraction, structural obfuscation and renaming, and injection of logical errors. We applied these erosion operators across five domains and quantified their effects on model performance using a publicly available language model. A two-way Analysis of Variance (ANOVA) revealed significant main effects of both domain and erosion method, as well as a significant interaction, indicating that the impact of semantic degradation depends jointly on how text is eroded and how domain-specific information is encoded. Logical error erosions proved especially damaging for code generation, whereas structural obfuscation most strongly impaired news and instruction tasks. Epistasis analysis of pairwise erosion unions showed that some combinations produced super-additive degradation while others exhibited compensatory effects. These domain-by-erosion profiles provide diagnostic insight into where multi-step large language model (LLM) pipelines are most likely to fail and suggest that robustness benchmarks should probe models along domain-specific vulnerability dimensions rather than relying on generic perturbations. Semantic erosion thus offers a principled tool for turning model failure into evidence about how language models structure and degrade meaning.

## 1 Introduction

When engaging with a text, our primary concern is typically the extent to which it can be understood. Why, then, should we concern ourselves with the removal of meaning in a systematic and quantifiable manner? The motivation arises from the rapid emergence and widespread deployment of artificial intelligence (AI), particularly in the form of large language models (LLMs). To rigorously evaluate their capacities, we must probe beyond scenarios where all relevant information is provided explicitly. Producing correct responses under fully specified conditions is a limited test of capability. More informative is the ability of models to operate under conditions of partial, ambiguous, or degraded input. To study this effectively, however, we require principled methods of withholding or obfuscating meaning that yield input whose level of degradation can itself be measured. Without such systematic control, the task reduces to a guessing game, and any quantitative metric derived from performance risks is ungrounded.

By contrast, the more qualitatively oriented traditions of the humanities, philosophy, and linguistics have long centered their inquiries on the “meaning of text.” This work provides a rich foundation of interpretive theories, but offers relatively little in the way of systematic or quantifiable approaches. The advent of large language models (LLMs) presents a new opportunity to move beyond purely interpretive accounts by testing how well such models “understand”^1^ meaning under controlled conditions. Programming problems provide a particularly effective testbed: they allow natural language prompts to be translated into executable code, whose functionality can then be objectively verified. While this approach necessarily entails domain knowledge of coding—as understanding in any context presupposes domain-relevant competence—it provides a clear framework for evaluating whether meaning survives after deliberate manipulations. The central challenge, however, lies not in verifying the code itself, but in developing a principled methodology for systematically removing meaning from the text in the first place. Consequently, we identified five key concepts to remove meaning from text, which we will discuss below, and we will test their effect and how their effect compounds in the experimental section.

### 1.1 Text erosion method 1: omitting key information and context removal

The impact of missing information on text comprehension has been rigorously documented through decades of discourse analysis and psycholinguistic research. Halliday and Hasan's seminal work on *Cohesion in English* (Halliday and Hasan, 1976) established that textual cohesion creates the property of “texture”—the capacity to interpret discourse as a unified whole rather than as disconnected sentences. Their taxonomy identified five cohesion types (reference, substitution, ellipsis, conjunction, and lexical cohesion), demonstrating that disrupting these ties significantly degrades comprehension.

Sanders, Spooren, and Noordman's cognitive framework for coherence relations (Sanders et al., 1992) advanced this line of inquiry, identifying four cognitive primitives (basic operation, polarity, source of coherence, and order). Their experimental work showed that when coherence relations are omitted, readers must expend greater cognitive effort and often fail to construct coherent mental representations. Similarly, Mann and Thompson's Rhetorical Structure Theory (RST) (Mann and Thompson, 1988) demonstrated that texts are organized hierarchically into nucleus-satellite relations, and that removing nuclei causes more severe comprehension breakdowns than omitting satellites.

Evidence from cognitive neuroscience strengthens these findings. Kim et al. (2012) showed that texts requiring bridging inferences activate the left middle temporal gyrus, whereas strongly coherent texts engage the dorsomedial prefrontal cortex, indicating distinct neural processing modes for degraded vs. intact discourse. Singer's validation model (Singer, 2013) and Graesser, Singer, and Trabasso's constructionist theory (Graesser et al., 1994) further demonstrated that missing causal information significantly slows comprehension and increases cognitive load, forcing readers from automatic into controlled processing.

Additional work on informativeness and discourse organization supports these observations. Giora's research on informativeness (Giora, 1988, 1997) established that discourse follows categorization principles, with missing informative content disrupting topic continuity. Similarly, studies on Gricean maxims (Grice, 1975) reveal that withholding relevant information or violating the maxim of quantity creates systematic comprehension failures. Developmental research confirms this sensitivity: even young children readily detect violations of conversational norms (Okanda et al., 2015).

### 1.2 Text erosion method 2: lexical substitution with near-synonyms

The linguistic impossibility of true synonymy and the comprehension effects of near-synonym substitution have been definitively established. Cruse (1986)'s *Lexical Semantics* provided a foundational account, arguing that “natural languages abhor absolute synonyms just as nature abhors a vacuum.” He distinguished cognitive synonyms from plesionyms (near-synonyms), showing that words appearing synonymous always differ in connotation, register, collocational preferences, or contextual usage.

Building on this, Edmonds and Hirst (2002) developed the most comprehensive computational model of near-synonymy. They identified four major types of variation: denotational (propositional, fuzzy, peripheral aspects), stylistic (dialect, register, formality), expressive (emotive and attitudinal), and structural (collocational, selectional, syntactic differences). Their clustered model of lexical knowledge demonstrates that near-synonyms share coarse-grained concepts but differ in fine-grained representation, making substitution a systematic source of semantic degradation.

Research on semantic drift confirms the instability of word meaning over time. Hamilton et al. (2016) distinguished cultural shifts from linguistic drift through processes such as subjectification, metaphorization, and grammaticalization. Such shifts prevent absolute synonymy, as words continually undergo narrowing, widening, amelioration, or pejoration. Corpus-based studies of collocational preferences (Kamiński, 2017) highlight these differences: for example, “strong coffee” is acceptable, whereas “powerful coffee” is not, despite the apparent synonymy of *strong* and *powerful*.

Psycholinguistic work further confirms comprehension effects. Studies show that semantically dissimilar substitutions disrupt comprehension more strongly than graphically similar ones, altering truth conditions, communicative impact, and processing difficulty (Priebe et al., 2012). Computational linguistics has extensively explored substitution effects, as documented in the ACL Anthology (Bird et al., 2008). Recent methods such as ParaLS (Qiang et al., 2023) and LexSubCon (Michalopoulos et al., 2021) combine contextual embeddings with lexical knowledge to better preserve meaning under substitution, yet even these models highlight the inherent fragility of semantic equivalence.

### 1.3 Text erosion method 3: increasing abstraction and generalizing language

The distinction between concrete and abstract language has long been central to psycholinguistic theory. Allan Paivio's dual coding framework (Paivio, 1990) remains foundational, showing that concrete words activate both verbal and nonverbal (imagery) representational systems, whereas abstract words primarily engage the verbal system. This dual coding advantage explains why concrete language is processed faster and more accurately across lexical decision, recognition memory, and recall tasks (Welcome et al., 2011).

Robust concreteness effects have been documented across numerous studies. Participants consistently classify concrete sentences more quickly than abstract ones, and abstract sentences are more frequently omitted in recall tasks. These findings replicate across languages including English, French, Chinese, and Italian, suggesting universal cognitive mechanisms (Gutchess and Indeck, 2009).

Schwanenflugel's context availability theory (Schwanenflugel et al., 1988) offers an alternative explanation. According to this account, abstract words are harder to process because they are supported by fewer contextual associations in memory. When abstract and concrete words are equated for context availability, concreteness effects often disappear, indicating that concrete words activate richer and broader verbal networks.

Neurocognitive research provides further evidence for distinct processing mechanisms. fMRI studies show that concrete words preferentially activate bilateral parietal lobes, left inferior frontal areas, the precuneus, and visual-perceptual regions including occipital and posterior temporal cortex. In contrast, abstract words elicit stronger activity in the left inferior frontal gyrus, superior temporal areas, and regions associated with semantic control (Kiehl et al., 1999; Kumar, 2016). Electrophysiological evidence corroborates these findings: abstract words elicit larger N400 responses, reflecting increased semantic processing demands, while concrete words evoke larger late positive components, potentially tied to mental imagery activation (Welcome et al., 2011).

### 1.4 Text erosion method 4: obfuscating structural clues and renaming

Research in both software engineering and historical linguistics highlights the importance of structural markers for comprehension. In programming, identifier naming has been shown to play a critical role. Binkley et al. (2013) demonstrated that identifier style (e.g., camelCase vs. underscore) significantly affects the speed and accuracy of code comprehension. Lawrie et al. (2007) compared single letters, abbreviations, and full-word identifiers, finding that descriptive names provide the best comprehension outcomes. Deissenboeck and Pizka (2006) introduced formal rules for well-formed identifiers, offering mathematical foundations for assessing identifier quality. Butler et al. (2009) further showed statistically significant associations between flawed identifiers and code quality issues. Interestingly, Hofmeister et al. (2019) reported that shorter identifiers often take longer to comprehend, as professional developers need more time to locate defects with abbreviated names. These findings converge on the principle that consistent, descriptive structural cues are essential for efficient comprehension in code.

Historical linguistics reveals strikingly parallel dynamics. Paul Saenger's *Space Between Words* (Saenger, 1997) traced how Irish scribes in the 7th–8th centuries introduced word spacing to make Latin more accessible as a second language. The transition from scriptura continua (continuous writing without spaces) to spaced text fundamentally transformed the neurophysiology of reading, enabling the historical shift from oral to silent reading. Parkes's *Pause and Effect* (Parkes, 2016) documented the parallel evolution of punctuation, which developed from rudimentary aids for inexperienced readers into sophisticated structural markers of meaning. Ancient Greek and Roman texts employed minimal punctuation, while medieval scribes introduced punctuation to resolve structural ambiguities.

Both domains—software engineering and historical linguistics—demonstrate that structural obfuscation significantly impedes comprehension. Just as poor identifier naming burdens code readers, the absence of spacing or punctuation once hindered textual interpretation.

### 1.5 Text erosion method 5: injecting logical errors

A final method of meaning erosion involves the deliberate injection of logical errors, disrupting coherence at the propositional level. Software engineering provides a systematic foundation for this practice. IEEE Standard 1044-2009 (IEEE, 2009) offers a definitive framework for classifying software anomalies, distinguishing defects, faults, and failures with attributes spanning type categories (data, interface, logic, description, syntax), mode categories (wrong, missing, extra), and effect categories (functionality, usability, security, performance). Beizer's classic *Software Testing Techniques* (Beizer, 2003) further articulated a four-level hierarchical classification covering requirements, features, structural defects, data, implementation, integration, and system architecture errors. Together, these frameworks formalize how injected logical inconsistencies can be described, categorized, and assessed.

Cognitive psychology research on coherence monitoring reveals striking parallels in natural language. Readers automatically track and validate textual consistency. Baker and Oakhill's work (e.g., Currie et al., 2021) shows that coherence monitoring includes detecting inconsistencies, nonwords, prior knowledge violations, and contradictions. O'Brien and Albrecht's “contradiction effect” (Myers et al., 1994) demonstrates that readers take significantly longer to process sentences that contradict earlier text, even when locally coherent, reflecting automatic validation processes.

Graesser and McNamara's Coh-Metrix system (Graesser and McNamara, 2011) measures over 200 indices of cohesion, language, and readability, showing that coherence breaks substantially increase reading times and impair memory. Their constructionist theory posits that readers engage in an ongoing “search after meaning,” with causal coherence playing a central role in early semantic processing (e.g., N400 ERP effects). Empirical studies confirm that coherence breaks extend reading times by 200–400 ms per sentence, with effects propagating into subsequent text. Eye-tracking data reveal longer fixations on inconsistent information, reduced recall for logically flawed passages, and impaired construction of situation models (Hakala and O'Brien, 1995; Metcalfe, 2017).

Taken together, the five methods described above—omission of key information and context, lexical substitution with near-synonyms, increased abstraction, structural obfuscation and renaming, and injection of logical errors—each draw on established research in discourse analysis, psycholinguistics, software engineering, or historical linguistics. Each targets a different dimension along which meaning can be degraded: cohesive and causal ties, fine-grained lexical distinctions, the concreteness–abstraction gradient, structural and formatting cues, and propositional consistency. This theoretical grounding provides a principled basis for treating these methods as controlled and complementary tools for eroding meaning, and for studying how their effects differ across domains and compound when combined.

### 1.6 Relation to existing robustness evaluation

A growing body of work evaluates Natural Language Processing (NLP) and LLM robustness through adversarial perturbations. Ribeiro et al.'s CheckList framework (Ribeiro et al., 2020) introduced behavioral testing inspired by software engineering, probing model capabilities through invariance tests and minimum functionality checks. PromptBench (Zhu et al., 2023) extended this to large language models, evaluating robustness against adversarial prompt perturbations at character, word, sentence, and semantic levels.

These approaches address a fundamentally different question than semantic erosion. Adversarial perturbation methods operate on the *surface form* of the input: they seek to break a model by swapping characters, injecting tokens, or rephrasing prompts while preserving the underlying meaning as much as possible. The object of study is the model's parsing robustness. Semantic erosion, by contrast, operates on the *meaning* of the text itself: it systematically removes or degrades the information content along theoretically defined dimensions. The object of our study is how different types of genuine meaning loss affect performance across domains. In adversarial testing, the meaning stays the same but the form changes; in semantic erosion, the meaning changes but the text remains well-formed. This distinction places the two approaches in different analytical spaces, addressing model robustness and meaning dependence, respectively.

## 2 Materials and methods

**Figure 1:**
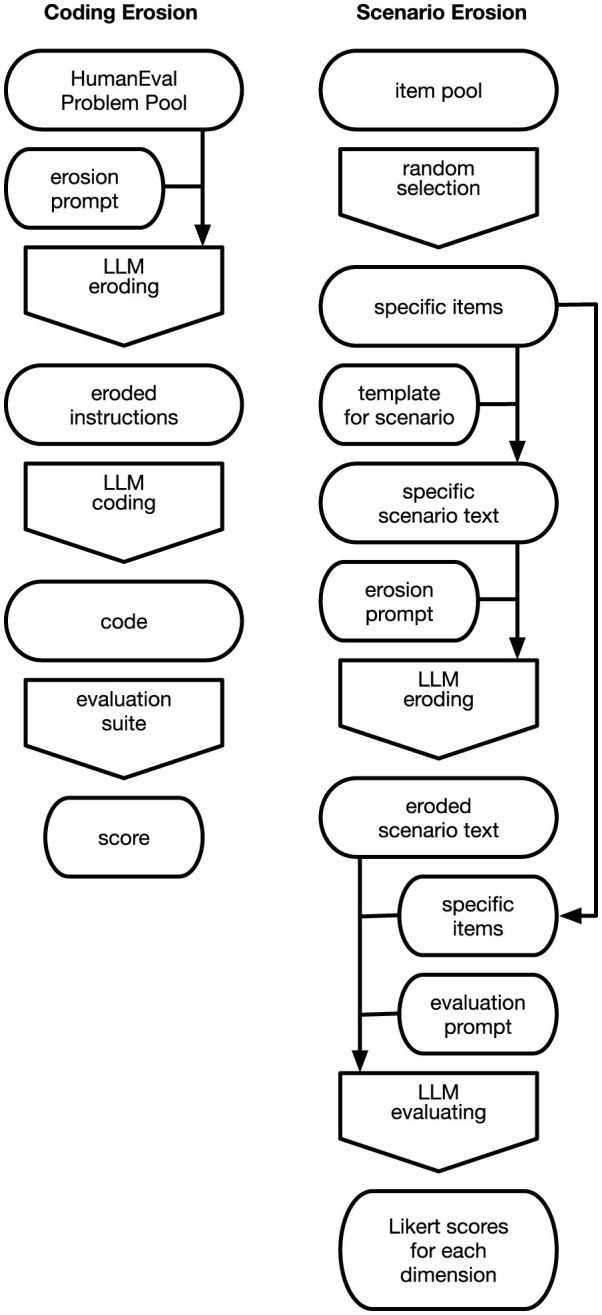
Overview of the two experimental pipelines. **(Left)** In the coding erosion pipeline, a HumanEval problem is eroded by an LLM and the resulting prompt is used to generate code, which is scored against the benchmark's test suite. **(Right)** In the scenario erosion pipeline, semantic dimensions are randomly sampled to generate a reference text, which is then eroded and evaluated by an LLM that attempts to recover each original dimension.

The experimental framework comprises two parallel pipelines, illustrated in Figure 1, applied across five domains (code generation, email, news, meeting summaries, and instructions) and five erosion methods (omission of key information and context, lexical substitution with near-synonyms, increased abstraction, structural obfuscation and renaming, and injection of logical errors). In the *coding erosion* pipeline, prompts from the HumanEval benchmark are subjected to one of the five erosion methods via an LLM, and the eroded prompt is then used to generate code whose correctness is verified against the benchmark's test suite. In the *scenario erosion* pipeline, a reference text is first generated from randomly sampled semantic dimensions, then eroded, and finally evaluated by an LLM that attempts to recover each original dimension from the eroded text. Both pipelines share the same five erosion operators and the same underlying language model, differing only in how inputs are constructed and outputs are scored. In addition to testing each erosion method individually, all pairwise sequential combinations of erosion methods were applied in both orders to quantify interaction effects (epistasis) across all five domains. The following subsections describe each component in detail.

### 2.1 Code generation and testing

The code generation task is based on the HumanEval benchmark (Chen et al., 2021), a collection of 164 Python programming problems, each consisting of a function signature, a docstring describing the task, and a set of unit-test assertions that verify correctness. Each prompt requires the model to complete a Python function body that satisfies all provided assertions.

To establish a baseline, each original HumanEval prompt was passed directly to the LLM without modification, and the generated code was executed against the benchmark's test suite. Performance was recorded as an overall pass/fail outcome together with a subscore indicating the fraction of individual assertions passed (e.g., 5/7). Each of the 164 problems was run for 5 replicates at baseline.

For the erosion conditions, each original prompt was first transformed by an LLM using one of the five erosion method prompts, producing an eroded version of the programming task description. This eroded prompt was then passed to the same LLM for code generation, and the resulting code was tested against the same unmodified HumanEval test suite. By comparing baseline and eroded performance on identical test assertions, the effect of each erosion method on code generation could be quantified directly.

### 2.2 Application domains not involving code

Four non-code application domains were used: email, news, instructions, and meeting summaries. For each domain, a set of semantic dimensions describing the content was defined, each containing a pool of approximately ten possible items. For example, a news article has these ten dimensions: subject organization or entity, location, time frame, primary actor, event type or action taken, cause or motivation, consequence or impact, information source, specific statistic or number, and next step or outcome.

Each dimension had a corresponding pool of items; for example, the location dimension in the news task listed: downtown Seattle, suburban Phoenix, rural Vermont, coastal Maine, Chicago's west side, Austin tech district, Portland metro area, Boulder county, Miami beach area, and Detroit industrial zone.

**Figure 2:**
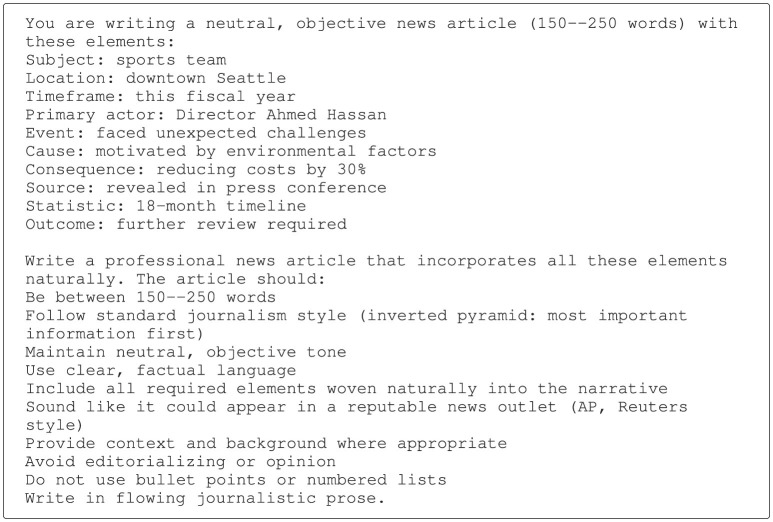
Example of a prompt for writing a professional news article.

To create a specific task, one item per dimension was randomly selected, and the selected items were inserted into a domain-specific prompt template together with a general task description (see Figure 2 for an example prompt).

**Figure 3:**
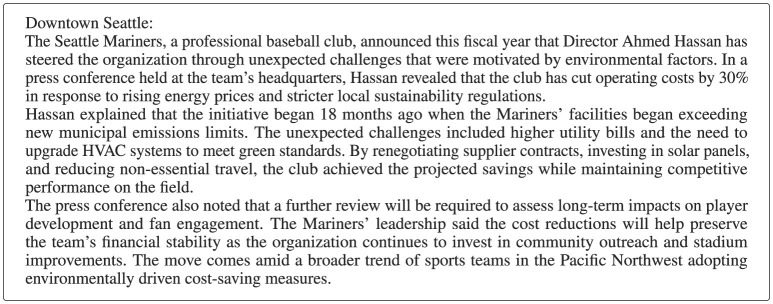
Example news article generated by the LLM from the prompt shown above. All ten semantic dimensions (subject, location, timeframe, primary actor, event, cause, consequence, source, statistic, and outcome) are woven into the text and serve as recoverable ground-truth items for the evaluation stage.

Such a prompt was then passed to an LLM to generate a reference text. Figure 3 shows an example news article produced from the prompt above.

Evaluation proceeded in two steps. First, the generated text (or its eroded version) was presented to an LLM together with one question per dimension, asking it to extract the relevant item. For the news article example, the following ten extraction questions were used:

1. What organization or entity is the article about?
2. Where did this take place?
3. When did this happen?
4. Who is the main person mentioned?
5. What happened or what action was taken?
6. What caused or motivated this event?
7. What was the impact or consequence?
8. What is the source of this information?
9. What specific number or statistic is mentioned?
10. What is the next step or outcome?

Second, each extracted answer was compared against the known ground truth (the item originally sampled for that dimension) by a separate LLM call that rated the agreement on a Likert scale from 1 (no match) to 10 (perfect match). The per-dimension scores were then averaged to produce a single performance score for each experiment. As with the code pipeline, a baseline was established by evaluating the original (uneroded) text, and the effect of each erosion method was measured as the change in score relative to this baseline. Each domain was run for 10 replicates per erosion condition.

All dimensions, their items, and the specific questions can be found in Supplementary material.

### 2.3 Erosion prompts

All five erosion methods were implemented as LLM-driven text transformations. For each method, a detailed system prompt instructs the model on the type of degradation to apply, provides concrete example transformations, and requires the output to remain grammatically well-formed. For instance, the context removal prompt directs the model to break causal chains, remove temporal connectives, delete conditional qualifiers, and eliminate bridging information, while preserving the overall structure of the text. The remaining four prompts follow the same pattern for their respective erosion types: the lexical substitution prompt targets vocabulary replacement with near-synonyms; the abstraction prompt replaces concrete details with more general terms; the structural obfuscation prompt removes or scrambles formatting cues, identifier names, and organizational markers; and the logical error prompt introduces contradictions or inconsistencies into the propositional content. In the code pipeline, the prompts additionally instruct the model to preserve function signatures and return statements, eroding only the natural-language description of the task. The exact text of all five erosion prompts is provided in Supplementary material (Section 1.1).

### 2.4 Models used

OpenAI's publicly available model gpt-oss:20b, a 20-billion-parameter open-weight language model, was used for all generation, erosion, and evaluation tasks. The model was run locally via Ollama using default inference settings. A single model was used deliberately across all pipeline stages (see Figure 1) to ensure internal consistency: any observed performance differences between domains or erosion methods can be attributed to the erosion itself rather than to differences between models. The choice of an open-weight model also ensures full reproducibility without dependence on proprietary API access. Manual spot checks of the model's evaluation outputs–both the code test results and the Likert-scale scoring of semantic dimensions in the non-code tasks–confirmed that the automated ratings aligned well with human judgment, supportin g the reliability of the LLM-as-judge approach for the narrow factual matching task used here.

The code for all experiments, analysis, and the resulting data can be found at: < made public upon acceptance of the manuscript>

## 3 Results

Three sets of experiments were conducted. First, the baseline performance of the model was measured in all five domains without any erosion applied. Second, each of the five erosion methods was applied individually, and the resulting performance was compared against the baseline. Third, all pairwise sequential combinations of erosion methods were applied to quantify their interaction effects (epistasis). The results of each set are presented in turn.

**Figure 4:**
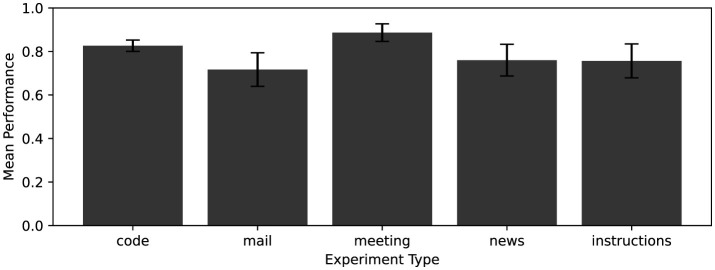
Baseline accuracy of the large language model in generating valid outputs for each of the five experimental domains: Code, Mail, Meeting, News, and Instructions. The 95% confidence errors are shown as error bars.

To establish the baseline, the model was tested on all five domains using the original, uneroded prompts: 5 replicates for each of the 164 coding problems, and 10 replicates for each of the four non-code tasks. The baseline performance for each domain is shown in Figure 4.

Each domain is not perfect, and while that is expected for the coding task, it shows that even other simple tasks can not be perfectly solved by the open accessible gpt-oss:20b model.

**Figure 5:**
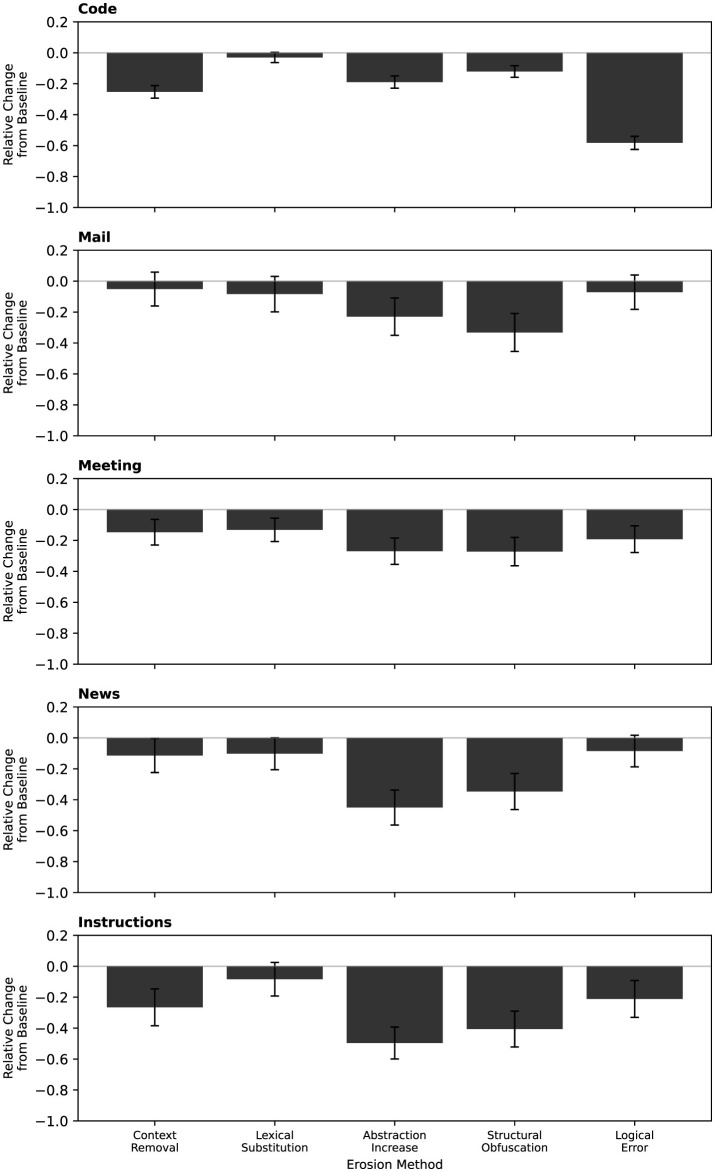
Relative performance change from baseline for five erosion methods: context removal, lexical substitutions, abstraction increase, structural obfuscation, and logical error introduction, across code generation, mail, meeting, news, and instruction tasks. Error bars show the 95% confidence interval.

Next, the effect of all five erosion models was tested. All generated text or code prompts were eroded using the five different erosion methods (context removal, lexical substitutions, abstraction increase, structural obfuscation, and logical error introduction), and their mean effect relative to the baseline can be seen in Figure 5.

A two-way ANOVA was conducted with *experiment type* (Code, Mail, Meeting, News, Instructions) and *erosion method* (Context Removal, Lexical Substitution, Abstraction Increase, Structural Obfuscation, Logical Error Introduction) as fixed factors, and model performance as the dependent variable. Both main effects were highly significant: experiment type, *F*(4, 7079) = 13.13, *p* = 1.18 × 10^−10^, η^2^ = 0.0067, and erosion method, *F*(5, 7079) = 95.04, *p* = 3.37 × 10^−97^, η^2^ = 0.0603. Critically, the interaction between experiment type and erosion method was also significant, *F*(20, 7079) = 13.94, *p* = 1.72 × 10^−46^, η^2^ = 0.0354, indicating that the relative impact of each erosion method differs across tasks. Although the model explains about 10% of the variance (*R*^2^ = 0.10), this pattern is consistent with the heterogeneity visible in Figure 5: code generation exhibits the largest degradation under logical-error and abstraction erosions, whereas communication-oriented tasks such as *Mail* and *Meeting* show milder and more uniform declines. These results show that erosion affects performance in a task-dependent manner, and not uniformly having the same effect.

### 3.1 *Post-hoc* pairwise comparisons

To identify which specific erosion methods and domains differ significantly, *post-hoc* pairwise comparisons were conducted using Tukey's Honestly Significant Difference (HSD) test (Tukey, 1949), which controls the family-wise error rate across all pairwise comparisons.

Across all domains, Logical Error erosion was the most damaging method, differing significantly from every other method (*p* < 0.001 in all cases). Structural Obfuscation was the second most harmful, significantly worse than Control, Lexical Substitution, and Logical Error. Lexical Substitution did not differ significantly from Control (*p* = 0.16), indicating that near-synonym replacement had minimal overall impact on performance. Context Removal and Abstraction Increase occupied an intermediate position and did not differ significantly from each other (*p* = 0.85).

Among domains, Meeting performance was significantly lower than Code, Mail, News, and Instructions (*p* < 0.01 in all cases). Code differed significantly from Instructions and Meeting, while Mail and News were statistically indistinguishable (*p*>0.99).

The most informative comparisons, however, were within individual domains, as these reveal the domain-specific erosion profiles. In Code, Logical Error erosion reduced performance far more than any other method (*p* < 0.001 against all others), consistent with the critical dependence of code on logical consistency. In Mail, only Structural Obfuscation stood out as significantly worse than all other methods (*p* < 0.05), while the remaining erosion types were statistically similar to one another. In News, Structural Obfuscation was again the most damaging method (*p* < 0.05 against all others except Abstraction Increase), and Abstraction Increase was significantly *less* harmful than all other erosion methods (*p* < 0.001). In Instructions, Abstraction Increase was the most damaging erosion (*p* < 0.05 against all others), and Structural Obfuscation also significantly reduced performance relative to Control (*p* < 0.001). In Meeting, Abstraction Increase, Logical Error, and Structural Obfuscation all differed significantly from Control (*p* < 0.05), reflecting a broader vulnerability across erosion types.

### 3.2 Interaction of erosion methods

The previous results show that the effect of erosion depends on the task, and thus, when considering the joint effect multiple erosion methods might have, we need to do this for each task independently. The interaction strength of two effects—their epistasis (*E*)—can be quantified using the following Equation 1:


$$\begin{array}{l} E=log\frac{W_{0}W_{AB}}{W_{A}W_{B}} \end{array}$$ (1)

**Figure 6:**
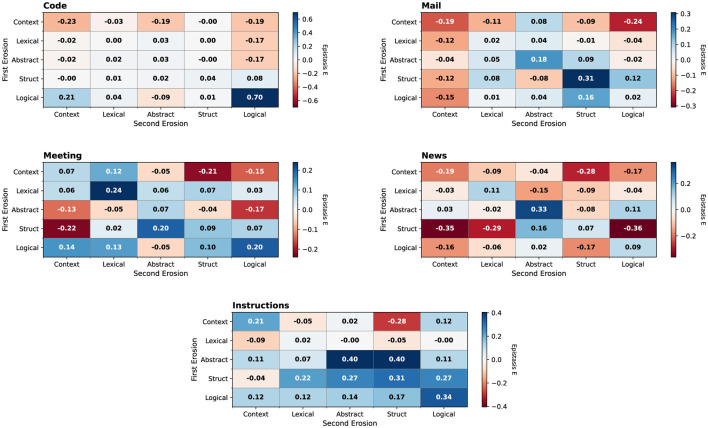
Epistasis matrices showing pairwise interaction effects between erosion methods for each experiment type. Negative values (red) indicate super-additive degradation, while positive values (blue) indicate mitigating or compensatory effects.

Here the strength of no erosion (*W*_0_) is compared to the joint effect of two erosions (*W*_*AB*_) and each individual effect (*W*_*A*_ and *W*_*B*_). Technically, the order of the effect in the calculation does not matter but in reality erosion is applied sequentially, one after the other. Thus all possible pairwise erosions, in each order, for all tasks were computed, and the results are seen in Figure 6.

## 4 Discussion

This study asked not only how much semantic degradation harms performance, but also where and how different types of erosion cause damage across domains. To address this, we introduced a framework for semantic erosion that unifies five theoretically grounded methods—omission of key information and context, lexical substitution with near-synonyms, increased abstraction, structural obfuscation and renaming, and the injection of logical errors—and applied it across five domains. Across tasks and degradation types, we found that different erosion operators preferentially target different kinds of information in the prompt, and that the tested model relies on these kinds of information to different degrees across domains. In other words, the impact of semantic erosion depends jointly on how text is degraded and how domain-specific information is encoded.

A two-way analysis of variance with domain and erosion method as factors showed significant effects of both factors, as well as a significant interaction. Baseline performance was imperfect in all domains, and the erosions produced heterogeneous drops in performance rather than the uniform penalty that would be expected from generic noise. Beyond single erosions, pairwise combinations were analyzed using an epistasis measure that compares the joint effect of two erosions against their joint individual effects. The results revealed that some combinations worsened performance more than expected, while others showed smaller-than-expected joint impact. To further unpack these patterns, we first examine how different domains depend on different parts of the prompt, and how this gives rise to distinct domain-specific erosion profiles.

### 4.1 Domain-specific erosion profiles

One way to understand domain dependence is to ask where the task-critical information is located in the prompt. The *post-hoc* comparisons reported in Section 3.1 provide the statistical foundation for the domain-specific profiles discussed below. In code, success depends on accurately representing conditions, invariants, and explicit relationships between variables and control structures (Back, 2006; Pennington, 1987; Wiedenbeck et al., 1993). Logical error erosions that introduce contradictions disturb these elements and make it difficult for the model to construct a coherent internal program that can be executed or verified. This behavior is consistent with software engineering taxonomies that treat logic faults as a major defect category (IEEE, 2009; Beizer, 2003).

News and instruction tasks, however, are more heavily dependent on discourse structure and event ordering. Here, performance relies on a situation model supported by temporal and causal links (Graesser et al., 1994; Singer, 2013). Structural obfuscation in the form of scrambling, disrupted ordering, or broken paragraphing weakens this situation model even when individual sentences remain grammatical and locally plausible. Mail and meeting domains combine aspects of both patterns: they contain logical and procedural content, but also rely strongly on contextual and social cues. Consequently, this mixed profile could explain why their erosion patterns fall between those of code and those of news or instructions.

These profiles also reflect differences in redundancy and error tolerance across tasks. Work on cohesion and multilevel discourse comprehension show that natural language texts often encode key information across multiple sentences and cohesive ties—including lexical repetition, anaphoric reference, and paraphrase—thus indicating that withholding relevant information or violating the maxim of quantity could lead to systematic comprehension failures (Grice, 1975; Giora, 1988; Graesser et al., 1994; Giora, 1997; Graesser and McNamara, 2011; Singer, 2013; Okanda et al., 2015; Hamilton et al., 2016). In such settings, models can sometimes compensate for lexical noise or mild logical inconsistencies by relying on remaining cues and prior knowledge. In contrast, code and tightly specified instructions are far less redundant, and even minor erosion can invalidate an entire solution or move it outside the scoring criterion. This lack of redundancy may explain why logical error erosions are especially destructive for code, while more redundant domains can partially repair them. Conversely, when global structure is disrupted in news and instruction tasks, redundancy is no longer accessible. Cues that would normally support reconstruction of ordering or causal structure are scattered or suppressed, and performance drops sharply even though individual propositions remain interpretable.

The epistasis analysis further clarifies how erosions interact rather than simply adding up. Negative epistasis values indicate cases where the joint effect of two erosions is worse than would be expected from the product of their individual effects. For example, in the news domain, applying structural obfuscation followed by context removal yields strongly negative epistasis (Figure 6), implying that disrupting discourse structure makes subsequent context loss particularly damaging. In contrast, some combinations of abstraction and structural obfuscation in instruction-like tasks show positive epistasis, which suggests partial compensation or ceiling effects in which additional degradation has limited marginal impact.

### 4.2 Diagnostics for multi-step pipelines

Domain-by-erosion profiles are not only descriptive; they also point to where real-world pipelines are most likely to fail when they process degraded input. In code generation pipelines, large language models translate natural language task descriptions into executable code (Wang et al., 2023). As shown in Section 4.1, our experiments show that logical error erosions in code-like tasks reduce performance far more than structurally unsound but logically intact prompt descriptions. This pattern is especially consequential in realistic multi-step workflows, where model outputs are repeatedly transformed. Each transformation step can be viewed as implementing an implicit erosion profile: some stages abstract away detail, others reorder or compress content, and some risk introducing small logical inconsistencies. In such settings, the domain-by-erosion map provides a diagnostic lens for reasoning about where pipelines are most likely to fail. If breakdowns concentrate in instruction-like tasks, stages that heavily obfuscate sequence and structure are natural suspects; if failures appear mainly in code-like tasks, stages that can inject logical contradictions deserve closer scrutiny.

This perspective also links LLM pipeline design to results in discourse processing, where omissions of causal information and violations of coherence relations are known to force readers into more demanding processing modes resulting in increased error rates (Sanders et al., 1992; Myers et al., 1994; Hakala and O'Brien, 1995; Singer, 2013; Currie et al., 2021). In information-seeking and procedural domains, the same patterns highlight structural and formatting changes as key failure modes (IEEE, 2009; Beizer, 2003). For systems that summarize news, condense meeting transcripts, or provide step-by-step guidance, enforcing minimal structural regularity and repairing obvious structural damage may be as important as filtering out factual noise.

Together, these findings suggest that practical systems should treat natural language specifications as potentially eroded along logical dimensions and actively guard against such erosion. In particular, systems should prioritize detection and repair of logical inconsistencies before asking the model to generate or modify code. Lightweight checks might include explicit consistency validation over numeric ranges and conditions, or clarification prompts that query the user when constraints appear incompatible. For code-like tasks, such logic-aware guardrails may offer more benefit than purely cosmetic reformatting of prompts, because they target the information that the domain relies on most heavily.

### 4.3 Implications for robustness benchmarks

These findings also have direct implications for how robustness benchmarks are designed. Benchmarks that apply only a single generic corruption, such as random lexical substitution, may give a misleadingly optimistic picture. Different erosion methods target different kinds of information, and domains differ in which information they rely on most. If only one erosion method is tested, a model may appear robust simply because the evaluation has avoided its weakest points.

Instead, we suggest organizing robustness benchmarks as a matrix of domains by erosion types and calibrating them so that each domain is probed along the dimensions it depends on most. The domain-dependent profiles observed here indicate which domain-erosion combinations are likely to reveal substantial degradation and which combinations mainly exercise already well-protected aspects of the model. In practice, this provides a starting point for designing more targeted stress tests that align with the pipeline vulnerabilities identified above.

### 4.4 Connections to human meaning making

Beyond engineering practice, the structure of these erosion effects also resonates with long-standing theories of how humans process degraded or incomplete language input. As discussed in Section 4.1, the strong effect of context removal on performance in several domains aligns with accounts of cohesion, coherence, and rhetorical structure, which emphasize the role of cohesive ties, discourse relations, and nucleus-satellite organization in supporting unified interpretation (Mann and Thompson, 1988; Sanders et al., 1992; Halliday and Hasan, 1976). It also aligns with work on informativeness and conversational norms, which shows that missing informative content or violations of the maxim of quantity lead to robust comprehension failures (Grice, 1975; Giora, 1988, 1997; Okanda et al., 2015). In our experiments, removing key contextual elements substantially degraded performance, even when sentence-level syntax remained intact, underscoring the role of context and informativeness in human discourse processing.

The lexical substitution method builds on work showing that genuine synonymy is rare and that near-synonyms differ in denotation, register, expressivity, and collocational profiles (Cruse, 1986; Edmonds and Hirst, 2002; Kamiński, 2017). Distributional analyses of semantic change likewise show that word meanings shift over time, further undermining assumptions of stable synonymy (Hamilton et al., 2016). Psycholinguistic studies report measurable comprehension costs when near-synonyms are substituted inappropriately (Priebe et al., 2012), and computational work on controlled paraphrasing confirms the difficulty of preserving fine-grained meaning under substitution (Bird et al., 2008; Michalopoulos et al., 2021; Qiang et al., 2023). Taken together, these results justify lexical substitution as a graded erosion method: the observed performance changes are consistent with the idea that such substitutions disrupt the model's ability to recover specific latent variables encoded in the prompt.

The abstraction increase erosion is grounded in dual coding theory and context availability accounts, which link concreteness to richer mental imagery and denser contextual associations (Schwanenflugel et al., 1988; Paivio, 1990; Gutchess and Indeck, 2009). Neurocognitive work shows distinct activation patterns for concrete and abstract words, with abstract terms often incurring higher semantic control demands (Kiehl et al., 1999; Welcome et al., 2011; Kumar, 2016). In our experiments, higher levels of abstraction led to poorer performance, particularly for code and instruction-like inputs, reinforcing the idea that concrete detail helps anchor interpretation for both humans and models.

Structural obfuscation and renaming connects software engineering findings on identifier naming and code readability with historical work on spacing and punctuation in written language. Programming studies show that descriptive identifiers and consistent naming styles support faster and more accurate comprehension, whereas abbreviated or inconsistent naming increases effort and defect rates (Deissenboeck and Pizka, 2006; Lawrie et al., 2007; Butler et al., 2009; Binkley et al., 2013; Hofmeister et al., 2019). Historical accounts of scriptura continua and the later introduction of word spacing and punctuation describe similar effects in written language, where structural markers transformed reading practices and accessibility (Saenger, 1997; Parkes, 2016). The structural erosion method abstracts these ideas into a unified manipulation of cues such as spacing, naming, and organizational markers. The corresponding performance drops provide evidence that, at least for this model class, the LLM relies heavily on such signals.

The logical error erosion method builds on both software anomaly taxonomies and psycholinguistic work on coherence monitoring. Formal classifications of defects and failures highlight logic errors as a distinct category with strong effects on functionality (IEEE, 2009; Beizer, 2003). In human reading, contradiction effects and coherence breaks reliably slow processing, impair memory, and reveal individual differences in coherence monitoring (Myers et al., 1994; Hakala and O'Brien, 1995; Graesser and McNamara, 2011; Metcalfe, 2017; Currie et al., 2021). As shown in Section 4.1, logical error erosions are especially harmful for code generation, whereas structural obfuscation is more harmful for news-like tasks, and as such illustrates how these theoretical constructs are expressed in LLM behavior at scale.

### 4.5 Limitations and future directions

Although the experiments span multiple domains and erosion types, the present study is necessarily constrained by a set of design choices. These constraints matter for how far the results can be generalized and for how they should inform future work. In this final subsection, we discuss the main limitations and outline directions for future work.

One set of limitations concerns the scope of domains, erosion operators, and models. The study focused on five common domains and a small, well-defined taxonomy of erosions chosen to capture major theoretical dimensions of meaning. Other codebases, writing genres, conversational styles, or instruction formats may exhibit different sensitivity patterns, especially in specialized or technical subdomains. Given the variety of domain-erosion interactions already observed here, extending both the range of domains and the erosion taxonomy—for example to multimodal prompts, longer documents, or adversarially designed perturbations—would likely reveal additional patterns rather than simply replicating the current ones. In the same vein, all experiments used a single publicly available model, gpt-oss:20b, for generation, erosion, and evaluation. This choice provides internal consistency but leaves open how architecture, size, and training data affect erosion sensitivity. Replicating the experimental design across a wider range of models would test the generality of the observed domain-erosion patterns and might reveal characteristic vulnerability profiles for different model families. These profiles, in turn, would help disentangle the contributions of architectural choices, training data composition, and fine-tuning. Finally, the non-code domains were incorporated through carefully constructed synthetic prompts. This design allowed precise control over latent variables but may differ from fully naturalistic data. Applying the same erosion methods to real-world emails, meeting transcripts, news articles, and instruction logs would help assess ecological validity and might uncover distributional effects that are not present in the designed items.

A second set of limitations arises from the way workflows and prompts are modeled. The present experiments isolate single-step transformations and pairwise combinations of erosions, and they treat prompts as given rather than manipulating the quality of user specifications themselves. In many deployed applications, however, LLMs are embedded in multi-step or agentic workflows in which intermediate outputs are repeatedly transformed, passed between tools, and reused as inputs. User prompts are also a major source of semantic erosion when key constraints are omitted, underspecified, or internally inconsistent. An open question is how local erosions at each stage of such workflows—whether introduced by tools or by prompts—accumulate across chains of transformations. Small degradations at each step may produce large cumulative meaning loss over long workflows. Extending the current framework to multi-stage settings, and evaluating interface designs that help users detect and repair prompt-level erosion before code or actions are executed—for example through structured input forms, validation of numeric ranges and constraints, or interactive clarification prompts—would allow these cumulative effects and their mitigations to be quantified directly.

A related limitation concerns erosion intensity. In the current design, each erosion method is treated as a categorical factor–applied or not applied–rather than as a continuous variable with adjustable severity. The LLM-driven erosion prompts instruct the model to apply a given type of degradation, but do not parameterize how strongly to erode. Intensity variation was instead captured through pairwise sequential combinations, which provide a discrete form of compounding. Introducing graded erosion–for example by instructing the model to erode “mildly” vs. “severely”–is a natural extension, but it also introduces a prompt engineering problem that is orthogonal to the framework itself: calibrating what “mild” and “severe” mean across erosion types and domains would require its own validation study.

A further direction is to more directly relate model vulnerabilities to human processing. In the present study, parallels between human and model behavior are inferred from existing work on software anomaly taxonomies and coherence monitoring, together with differences in how erosion types affect LLM performance across domains. A natural extension is to apply matched erosion schemes to human readers and LLMs and compare their robustness profiles, identifying where their sensitivity to logical errors and structural obfuscation converges and where it diverges. Such experiments could build on the more naturalistic settings outlined above by using shared materials for both humans and models. At the same time, although the analyses here emphasize domain specificity, certain erosion patterns may generalize across domains at a more abstract level as functions of redundancy, structural dependence, or logical coupling. A next step would be to analyze these relationships more formally and to test whether erosion can be turned into a systematic tool for mapping the computational phenotype of language models, thus characterizing not only how well they perform but also how and where they fail.

Despite these limitations, our experiments and subsequent analyses show that semantic erosion can be defined, implemented, and quantified in a systematic way, and that different erosions exploit distinct vulnerabilities in different domains. Because the erosion operators are grounded in established theories of cohesion, lexical semantics, abstraction, structural marking, and logical coherence, the study provides both a conceptual and a practical basis for designing more robust LLM pipelines, benchmarks, and training regimes. Semantic erosion thus offers a way to turn model failure into evidence, using breakdowns in performance to reveal how language models structure and degrade meaning. In that sense, our main contribution is not only the specific erosion patterns we define, but the demonstration that they can be engineered and reused as a general framework for probing, comparing, and improving language model output.

## 5 Conclusions

We presented a framework for systematically eroding and quantifying meaning in text, grounded in five theoretically motivated methods drawn from discourse analysis, psycholinguistics, and software engineering. Applied across five domains–code generation, email, news, meeting summaries, and instructions–the framework revealed that the impact of semantic degradation is neither uniform nor random: different erosion methods target different kinds of information, and different domains depend on these kinds of information to different degrees. Logical error erosions proved most damaging for code, structural obfuscation most strongly impaired news and instruction tasks, and pairwise combinations of erosions exhibited both super-additive and compensatory interaction patterns. These domain-by-erosion profiles provide actionable diagnostics for identifying where LLM pipelines are most vulnerable and suggest that robustness benchmarks should probe models along domain-specific dimensions rather than relying on generic perturbations. More broadly, the framework demonstrates that controlled removal of meaning can serve as a general tool for characterizing how language models process, rely on, and ultimately lose the information encoded in their inputs.

## Data Availability

The code for all experiments, analysis, and the resulting data are publicly available at https://osf.io/48tcx.

## Footnotes

1 We place terms such as *meaning* and *understanding* in quotation marks, as they are extremely hard to define for humans, let alone LLMs. Nevertheless, we assume the reader has sufficient intuition about these concepts without requiring exact definitions, which in our context are not essential.
