# Supplementary material for: How to systematically and quantifiably remove meaning?
Source: Front Artif Intell. 2026 May 13;9:1783410. doi: 10.3389/frai.2026.1783410 (PMC13212427; doi:10.3389/frai.2026.1783410)
Supplement: Supplementary file 1 [file Data_Sheet_1.pdf]

Supplementary Material

1 SUPPLEMENTARY DATA

1.1 Erosion Prompts

The following are the complete system prompts used to transform text according to each erosion method. These prompts were provided to a language model as system instructions, with the original text provided as user input.

1.1.1 Erosion Method 1: Omitting Key Information and Context Removal

```
You are a text transformation assistant. Your task is to remove key
↪  information and context from the provided text. Consider these
↪  different approaches as examples of how meaning erosion through
↪  omission might occur:

Possible erosion patterns through omission:

Causal relationship removal might look like:
- Breaking apart cause-and-effect chains
- Removing words such as: because, therefore, since, thus, consequently,
↪  as a result
- Separating linked statements into isolated facts
- Example transformation: The function returns null because the input is
↪  invalid → The function returns null. The input is invalid.

Temporal/sequential context removal might involve:
- Eliminating time-based connectives: after, before, when, while, during,
↪  then, next, first, finally
- Removing order dependencies between steps
- Example transformation: First initialize the array, then populate it
↪  with values → Initialize the array. Populate it with values.

Bridging information removal could include:
- Deleting explanatory connections between concepts
- Removing clarifying phrases: which means, this allows, in other words,
↪  that is
- Eliminating intermediate reasoning steps
- Example transformation: Set the flag to true. This will enable debugging
↪  mode. Run the test. → Set the flag to true. Run the test.

Constraint and condition removal might manifest as:
- Deleting conditional qualifiers: if, unless, only when, except, provided
↪  that
- Removing boundary specifications and scope limitations
- Example transformation: Process only positive integers less than 1000 →
↪  Process integers

Purpose and goal removal could appear as:
- Eliminating purpose indicators: in order to, so that, for the purpose
↪  of, to achieve
- Removing outcome specifications
```

```
- Example transformation: Sort the list in ascending order to optimize the
↪   search algorithm → Sort the list in ascending order

Apply one or more of these erosion patterns to the text, selecting the
↪   approach that most naturally fits the content. Maintain grammatical
↪   correctness while removing information that provides context,
↪   relationships, or clarification.

Output the transformed text. Keep the structure of the question the same.
↪   Preserve relevant formating instructions! Do not change the name and
↪   format of the function call or return line, only change the
↪   description of the functon between def and return!
```

1.1.2 Erosion Method 2: Lexical Substitution with Near-Synonyms

```
You are a text transformation assistant. Your task is to perform lexical
↪   substitution by replacing words with near-synonyms that subtly alter
↪   meaning. Consider these patterns of semantic drift through
↪   substitution:

Possible substitution patterns:

Register shifts might manifest as:
- Replacing formal terms with casual equivalents or vice versa
- Substituting technical vocabulary with everyday language
- Example transformation: The algorithm exhibits optimal performance → The
↪   algorithm shows good performance
- Example transformation: Fix the bug → Rectify the defect

Connotational drift could involve:
- Replacing neutral terms with slightly charged alternatives
- Substituting words that have similar denotation but different emotional
↪   weight
- Example transformation: The process terminated → The process ended
- Example transformation: Modify the settings → Alter the settings

Collocational mismatches might appear as:
- Using words that mean roughly the same but don't typically pair with
↪   surrounding words
- Breaking established word partnerships
- Example transformation: Make a decision → Do a decision
- Example transformation: Strong coffee → Powerful coffee

Scope alterations could include:
- Replacing specific terms with broader ones or vice versa
- Substituting words that shift the range of reference
- Example transformation: Calculate the sum → Compute the total
- Example transformation: Store the data → Keep the information

Semantic granularity changes might look like:
- Replacing precise technical terms with approximate equivalents
- Substituting words that blur fine distinctions
- Example transformation: Iterate through the array → Loop over the list
- Example transformation: Initialize the variable → Start the variable
```

```
Apply these substitution patterns throughout the text, choosing
↪  near-synonyms that create subtle semantic drift while maintaining
↪  grammatical correctness. The goal is to preserve surface readability
↪  while gradually shifting meaning through accumulated small changes.

Output the transformed text. Keep the structure of the question the same.
↪  Preserve relevant formating instructions! Do not change the name and
↪  format of the function call or return line, only change the
↪  description of the functon between def and return!
```

1.1.3 Erosion Method 3: Increasing Abstraction and Generalizing Language

```
You are a text transformation assistant. Your task is to increase
↪  abstraction and generalize language in the provided text. Consider
↪  these patterns of how concrete details might be replaced with abstract
↪  expressions:

Possible abstraction patterns:

Specific to general transformations might manifest as:
- Replacing concrete numbers with vague quantifiers
- Substituting specific items with category names
- Example transformation: Insert 5 elements into the array → Insert some
↪  elements into the collection
- Example transformation: Use a for-loop → Use a control structure

Action to process conversions could involve:
- Replacing concrete verbs with abstract ones
- Substituting specific operations with general processes
- Example transformation: Click the submit button → Interact with the
↪  interface element
- Example transformation: Concatenate the strings → Combine the data

Object to concept shifts might appear as:
- Replacing tangible items with abstract ideas
- Moving from instances to classes
- Example transformation: Store the username and password → Store the
↪  credentials
- Example transformation: The Python interpreter → The execution
↪  environment

Measurement to quality transformations could include:
- Replacing precise metrics with qualitative descriptions
- Substituting exact values with relative terms
- Example transformation: Wait 500 milliseconds → Wait briefly
- Example transformation: Allocate 1024 bytes → Allocate sufficient memory

Method to approach generalizations might look like:
- Replacing specific techniques with broad strategies
- Substituting concrete steps with abstract phases
- Example transformation: Use binary search to find the element → Apply an
↪  efficient search strategy
- Example transformation: Validate email format using regex → Perform
↪  input validation
```

```
Apply these abstraction patterns throughout the text, replacing concrete
↪  and specific language with abstract and general alternatives. The goal
↪  is to maintain grammatical correctness while reducing the precision
↪  and tangibility of the description.

Output the transformed text. Keep the structure of the question the same.
↪  Preserve relevant formating instructions! Do not change the name and
↪  format of the function call or return line, only change the
↪  description of the functon between def and return!
```

1.1.4 Erosion Method 4: Obfuscating Structural Clues and Renaming

```
You are a text transformation assistant. Your task is to obfuscate
↪  structural clues and rename identifiers in the provided text. Consider
↪  these patterns of how structural clarity might be degraded:

Possible obfuscation patterns:

Identifier renaming might manifest as:
- Replacing descriptive names with generic or meaningless labels
- Substituting full words with single letters or abbreviations
- Example transformation: userAge → x
- Example transformation: calculateTotalPrice → func1

Reference ambiguity could involve:
- Replacing specific referents with vague pronouns
- Using unclear anaphoric references
- Example transformation: Store the result in the cache variable → Store
↪  it in that
- Example transformation: The function defined above → The thing mentioned
↪  earlier

Structural marker removal might appear as:
- Eliminating organizational indicators
- Removing hierarchical cues and section markers
- Example transformation: Step 1: Initialize. Step 2: Process. Step 3:
↪  Return → Initialize. Process. Return.
- Example transformation: First method: validate input → validate input

Relationship obscuration could include:
- Removing explicit connections between components
- Eliminating cross-references and links
- Example transformation: As shown in the previous function → As shown
↪  somewhere
- Example transformation: This relates to the configuration discussed
↪  earlier → This relates to something

Naming consistency disruption might look like:
- Using multiple different terms for the same concept
- Varying terminology without pattern
- Example transformation: The array is initialized. The array is
↪  populated. The array is sorted. → The array is initialized. The list
↪  is populated. The collection is sorted.
- Example transformation: The server processes requests → The server
↪  processes requests, then the host handles queries
```

```
Apply these obfuscation patterns throughout the text, degrading structural
↪ clarity while maintaining basic grammatical correctness. The goal is
↪ to make relationships and references harder to track while keeping
↪ individual statements parseable.

Output the transformed text. Keep the structure of the question the same.
↪ Preserve relevant formating instructions! Do not change the name and
↪ format of the function call or return line, only change the
↪ description of the functon between def and return!
```

1.1.5 Erosion Method 5: Injecting Logical Errors

```
YYou are a text transformation assistant. Your task is to inject logical
↪ errors and inconsistencies into the provided text. Consider these
↪ patterns of how logical coherence might be disrupted:

Possible error injection patterns:

Causal inconsistencies might manifest as:
- Introducing effects that don't follow from their stated causes
- Creating circular reasoning or broken logical chains
- Example transformation: If x is greater than 5, return true → If x is
↪ greater than 5, return false
- Example transformation: Increment the counter to track iterations →
↪ Decrement the counter to track iterations

Conditional contradictions could involve:
- Making conditions and their consequences misaligned
- Introducing mutually exclusive requirements
- Example transformation: Process all even numbers → Process all even
↪ numbers that are odd
- Example transformation: Continue while the list is not empty → Continue
↪ while the list is full

Sequential disruptions might appear as:
- Placing dependent steps before their prerequisites
- Reversing necessary orderings
- Example transformation: Initialize the variable then declare it → Use
↪ the variable then declare it
- Example transformation: Open the file, write data, close the file →
↪ Write data, close the file, open the file

Boundary violations could include:
- Introducing off-by-one errors
- Creating impossible ranges or limits
- Example transformation: Iterate from 0 to array length - 1 → Iterate
↪ from 0 to array length
- Example transformation: Check if value is between 1 and 10 → Check if
↪ value is between 10 and 1

State inconsistencies might look like:
- Having objects in contradictory states simultaneously
- Violating state transition rules
```

```
- Example transformation: The empty list contains three elements → The
↪  empty list contains three elements
- Example transformation: After sorting in ascending order, the largest
↪  element is first → After sorting in ascending order, the largest
↪  element is first

Apply these error injection patterns sparingly throughout the text,
↪  introducing logical flaws that create inconsistencies while
↪  maintaining surface-level grammatical correctness. The goal is to
↪  corrupt the logical structure while keeping individual statements
↪  syntactically valid.

Output the transformed text. Keep the structure of the question the same.
↪  Preserve relevant formating instructions! Do not change the name and
↪  format of the function call or return line, only change the
↪  description of the functon between def and return!
```

1.2 Generation Method for None-Coding Tasks

Each task (email, news, instructions, and meeting summary) had 10 dimensions, and each dimension various items to select from. The following sections list each dimension, with its corresponding tasks, followed by the template for the prompt.

Randomly selected items were then used to generate the following prompt, which also included more specific instructions, narrowing the task, towards the end. The variables indicated by { variable } were replaced with the specific items characterizing the task (ground truth).

1.2.1 Mail/Email

- Sender: David Chen, Sarah Johnson, Ming Li, Carlos Rodriguez, Jessica Brown, Ahmad Hassan, Emily Davis, Ryan O'Brien, Priya Patel, Marcus Williams
- Recipient: Dr. Jennifer Smith (supervisor), Mike Chen (friend), Customer Service (support), Mom (parent), Coach Martinez (team leader), Professor Lee (instructor), Lisa Park (roommate), IT Support (technical), Dr. Ahmed (client), Grandpa Joe (grandparent)
- Emotional state: worried, frustrated, apologetic, confused, urgent, grateful, disappointed, excited, cautious, embarrassed
- Main issue: equipment malfunction, scheduling conflict, missing item, deadline concern, misunderstanding to clarify, permission request, error in document, unexpected expense, change in plans, technical difficulty
- Specific item: laptop, report, keys, presentation, invoice, reservation, package, files, equipment, tickets
- Location: conference room B, parking lot, main office, break room, storage unit, meeting point, lab 3, downtown branch, east entrance, second floor
- Timeframe: by end of day, tomorrow morning, within the week, before Friday, ASAP, next Monday, in two hours, by month end, before the meeting, when possible
- Requested action: please confirm, could you check, need approval, please advise, can we reschedule, please send, could you clarify, need your input, please call me, can you help
- General context: work project, family event, customer complaint, academic assignment, maintenance issue, travel arrangement, medical appointment, financial matter, team coordination, personal favor

- Relationship type: professional, casual, formal request, family, recreational, academic, domestic, troubleshooting, business, personal

Generation prompt: You are writing a detailed email (150-250 words) with these characteristics:

- From: sender
- To: {recipient name} (their {recipient role})
- The sender is feeling {emotional state}
- General context: {context}
- Main issue: {issue}
- Must mention: a {item} and {location}
- Needs something done {timeframe}
- Requesting: {action}

Write a natural, realistic email that incorporates all these elements organically. The email should:

- Be between 150-250 words
- Include appropriate greeting and closing
- Provide enough context and detail to reach the target length
- Sound like authentic human communication
- Naturally weave in all required elements without making them feel forced
- Match the emotional tone throughout

Do not number or list the elements - incorporate them naturally into flowing prose.

1.2.2 News Article

- Subject organization: local school, tech company, city council, research team, hospital system, transportation authority, environmental group, sports team, cultural institution, small business
- Location: downtown Seattle, suburban Phoenix, rural Vermont, coastal Maine, Chicago's west side, Austin tech district, Portland metro area, Boulder county, Miami beach area, Detroit industrial zone
- Timeframe: yesterday afternoon, early this morning, late last week, over the weekend, on Tuesday, during the past month, last quarter, this fiscal year, since January, throughout the summer
- Primary actor: Mayor Linda Rodriguez, Dr. James Chen, CEO Sarah Mitchell, Coach Mike Thompson, Director Ahmed Hassan, Professor Emily Park, Captain David Liu, Principal Maria Garcia, Commissioner Robert Johnson, Founder Jessica Lee
- Event type: announced a new initiative, reported record numbers, faced unexpected challenges, celebrated a major milestone, responded to community concerns, launched a pilot program, discovered significant findings, experienced technical difficulties, reached a settlement, revealed expansion plans
- Cause/motivation: due to funding increases, following community feedback, after safety concerns, in response to demand, because of regulatory changes, driven by research findings, prompted by recent events, as part of strategic planning, following a thorough review, motivated by environmental factors
- Consequence/impact: affecting over 5,000 residents, creating 200 new jobs, reducing costs by 30%, improving service quality, raising safety concerns, delaying the project timeline, exceeding expectations, requiring additional resources, changing local regulations, setting a regional precedent

- Information source: according to official statements, based on internal documents, per spokesperson confirmation, as reported in quarterly filings, according to eyewitness accounts, confirmed by department records, revealed in press conference, stated in public hearing, disclosed in annual report, verified by independent analysis
- Statistic/number: 23% increase, $2.4 million investment, 450 participants, 18-month timeline, 3,200 square feet, 87% approval rating, 40-person team, 15-year partnership, $850,000 budget, 62% completion rate
- Next step/outcome: implementation begins next quarter, public comment period opens, further review required, partnership agreement finalized, pilot phase completed successfully, expansion approved unanimously, opposition groups mobilizing, referendum scheduled for November, interim measures adopted, stakeholders remain divided

Generation prompt: You are writing a neutral, objective news article (150-250 words) with these elements:

- Subject: subject
- Location: location
- Timeframe: timeframe
- Primary actor: primary_actor
- Event: event_type
- Cause: cause
- Consequence: consequence
- Source: source
- Statistic: statistic
- Outcome: outcome

Write a professional news article that incorporates all these elements naturally. The article should:

- Be between 150-250 words
- Follow standard journalism style (inverted pyramid: most important information first)
- Maintain neutral, objective tone
- Use clear, factual language
- Include all required elements woven naturally into the narrative
- Sound like it could appear in a reputable news outlet (AP, Reuters style)
- Provide context and background where appropriate
- Avoid editorializing or opinion

Do not use bullet points or numbered lists - write in flowing journalistic prose.

1.2.3 Meeting Summary

- Participants: Sarah Chen (VP Operations), Mark Thompson (Finance), Lisa Rodriguez (HR), Dr. James Park (Lead Researcher), Emily Wu (Data Analyst), Michael Brown (Lab Manager), Karen Johnson (Marketing Director), Tom Anderson (Sales Lead), Jessica Lee (Product), David Martinez (CEO), Amanda Singh (CTO), Robert Kim (Legal Counsel), Professor Linda Green (Chair), Dr. Ahmed Hassan (Member), Dr. Maria Santos (Member), Jennifer White (Project Manager), Chris Taylor

(Engineering), Patricia Moore (QA), Kevin O'Brien (Board President), Susan Clark (Treasurer), Daniel Lee (Secretary), Rachel Adams (Creative Director), Jason Miller (Account Manager), Nicole Turner (Designer), Dr. William Chen (Medical Director), Laura Davis (Nursing Supervisor), Frank Wilson (Admin), Michelle Garcia (Community Liaison), Brian Jackson (Development), Angela Martinez (Outreach)

- Meeting purpose: quarterly budget review, product launch planning, crisis response coordination, strategic planning session, performance evaluation discussion, policy revision meeting, project kickoff, post-mortem analysis, stakeholder alignment, resource allocation review
- Key decision: approved the new budget allocation, decided to delay the launch, adopted revised safety protocols, selected the vendor proposal, agreed to restructure the team, voted to pursue the merger, authorized additional funding, implemented new workflow procedures, declined the partnership offer, committed to the expansion plan
- Primary action item: draft implementation timeline, conduct stakeholder interviews, prepare cost-benefit analysis, update compliance documentation, schedule follow-up meetings, revise project specifications, coordinate with external partners, develop training materials, audit current processes, create communication plan
- Responsible person: Mark Thompson, Emily Wu, Tom Anderson, Amanda Singh, Dr. Ahmed Hassan, Chris Taylor, Susan Clark, Jason Miller, Laura Davis, Brian Jackson
- Deadline: by end of Q2, within two weeks, before next board meeting, by Friday, end of fiscal year, within 30 days, by mid-month, next quarterly review, end of pilot phase, before annual conference
- Budget/resources: $125,000 allocation, 3 additional staff members, new software licenses, external consulting budget, equipment upgrade funds, training budget of $45,000, facility expansion resources, marketing spend increase, R&D investment of $200,000, operational reserves
- Concern/objection: timeline feasibility concerns, budget constraint worries, staff capacity limitations, regulatory compliance questions, technical implementation risks, stakeholder resistance anticipated, resource availability issues, competitive market pressures, data security considerations, change management challenges
- Next meeting: scheduled for two weeks, tentatively set for next month, to be determined based on progress, planned for end of quarter, set for following Tuesday, scheduled after vendor presentations, proposed for mid-cycle review, to reconvene in 30 days, arranged for next board session, planned following pilot completion
- Next step/outcome: consensus reached on path forward, phase one approved with conditions, further analysis required, unanimous approval granted, pilot program green-lighted, deferred pending additional data, conditional approval with milestones, split decision requiring follow-up, full endorsement achieved, revised proposal requested

Generation prompt: You are writing a professional meeting summary (150-250 words) with these elements:

- Participants: participants
- Meeting purpose: meeting_purpose
- Key decision: key_decision
- Action item: action_item
- Responsible person: responsible_person

- Deadline: deadline
- Budget/Resources: budget_resource
- Concern raised: concern
- Next meeting: next_meeting
- Outcome: outcome

Write a clear, professional meeting summary that incorporates all these elements naturally. The summary should:

- Be between 150-250 words
- Follow standard meeting minutes/summary format
- Use professional, objective tone
- Clearly identify who attended, what was discussed, and what was decided
- Include all required elements woven naturally into the narrative
- Provide enough detail for someone who didn't attend to understand what happened
- Organize information logically (attendees, purpose, discussion, decisions, next steps)
- Use clear, concise language appropriate for business documentation

Do not use bullet points or numbered lists - write in flowing professional prose that would be appropriate for corporate meeting minutes.

1.2.4 Procedural Instructions

- Task name: replacing a bicycle tire, baking sourdough bread, installing a ceiling fan, troubleshooting WiFi connection, preparing homemade pasta, changing engine oil, setting up a fish aquarium, repairing a leaky faucet, configuring email encryption, building a raised garden bed
- Primary tool: adjustable wrench, stand mixer, wire stripper, network cable tester, rolling pin, oil filter wrench, water testing kit, pipe wrench, PGP key generator, circular saw
- Key material: inner tube and tire levers, active sourdough starter, electrical wire nuts, ethernet cable, 00 flour, synthetic 5W-30 oil, biological filter media, plumber's tape, public key certificate, pressure-treated lumber
- Duration: 30-45 minutes, 6-8 hours total, 1-2 hours, 15-20 minutes, 2-3 hours including rest time, 45 minutes to 1 hour, 3-4 hours for initial setup, 20-30 minutes, 10-15 minutes, half day project
- First step: remove the wheel from the bike frame, feed and activate your starter 8 hours before, turn off power at the circuit breaker, unplug the router and modem, measure and mix flour with eggs, warm up the engine for 5 minutes, rinse the tank thoroughly with clean water, shut off the water supply valve, generate a new key pair on your device, mark and measure the corner posts
- Critical step: ensure the tire bead seats properly in the rim, don't let the dough over-proof, connect the ground wire to the metal box, test each cable end for continuity, knead until the dough passes the windowpane test, replace the drain plug carefully to avoid stripping, cycle the tank for 4-6 weeks before adding fish, tighten the compression fitting evenly, never share your private key, check that all corners are perfectly square
- Safety warning: check for rim damage before inflating, watch for steam when opening the oven, verify power is off with a voltage tester, avoid pinching fingers in cable crimper, keep raw eggs away from

other ingredients, hot oil can cause severe burns, never use soap as it can harm fish, protect eyes from splashing water, backup your key in a secure location, wear safety glasses when cutting

- Expected result: tire should hold pressure without slow leaks, crusty exterior with open crumb structure, fan should run smoothly on all speed settings, consistent internet connection restored, silky pasta with slight bite when cooked, oil should be clear amber color, clear water with stable parameters, no drips from the faucet, ability to send and receive encrypted messages, level, stable structure
- Common mistake: pinching the tube when installing the tire, adding too much flour during shaping, reversing the hot and neutral wires, not testing cables before installation, adding water instead of eggs, overtightening the oil filter, adding too many fish too quickly, using the wrong size washer, using a weak passphrase, not accounting for wood expansion
- Prerequisite: basic familiarity with bike components, understanding of yeast fermentation, ability to identify electrical wires, basic networking knowledge, experience with basic dough techniques, access to appropriate disposal for old oil, understanding of nitrogen cycle, knowing your home's plumbing layout, basic understanding of public key cryptography, ability to use power tools safely

Generation prompt: You are writing clear, detailed instructions (150-250 words) for completing a task with these elements:

- Task: task_name
- Primary tool: primary_tool
- Key material: material
- Duration: duration
- First step: first_step
- Critical step: critical_step
- Safety warning: safety_warning
- Expected result: expected_result
- Common mistake: common_mistake
- Prerequisite: prerequisite

Write comprehensive procedural instructions that incorporate all these elements naturally. The instructions should:

- Be between 150-250 words
- Follow a logical, step-by-step flow
- Use clear, imperative language ("do this," "check that")
- Include all required elements woven naturally into the procedure
- Provide enough detail for someone to successfully complete the task
- Sound like a how-to guide or tutorial
- Balance completeness with conciseness
- Include context where helpful

Do not use bullet points or numbered lists - write in flowing instructional prose that guides the reader through the process naturally, similar to a detailed recipe or tutorial article.
